# Histological and serological features of acute liver injury after SARS-CoV-2 vaccination

**DOI:** 10.1016/j.jhepr.2022.100605

**Published:** 2022-10-13

**Authors:** Greta Codoni, Theresa Kirchner, Bastian Engel, Alejandra Maria Villamil, Cumali Efe, Albert Friedrich Stättermayer, Jan Philipp Weltzsch, Marcial Sebode, Christine Bernsmeier, Ana Lleo, Tom JG. Gevers, Limas Kupčinskas, Agustin Castiella, Jose Pinazo, Eleonora De Martin, Ingrid Bobis, Thomas Damgaard Sandahl, Federica Pedica, Federica Invernizzi, Paolo Del Poggio, Tony Bruns, Mirjam Kolev, Nasser Semmo, Fernando Bessone, Baptiste Giguet, Guido Poggi, Masayuki Ueno, Helena Jang, Gülsüm Özlem Elpek, Neşe Karadağ Soylu, Andreas Cerny, Heiner Wedemeyer, Diego Vergani, Giorgina Mieli-Vergani, M. Isabel Lucena, Raul J. Andrade, Yoh Zen, Richard Taubert, Benedetta Terziroli Beretta-Piccoli

**Affiliations:** 1 Università della Svizzera Italiana, Facoltà di Scienze Biomediche, Lugano, Switzerland; 2 Dept. Gastroenterology, Hepatology and Endocrinology, Hannover Medical School, Hannover, Germany; 3 HIBA, Buenos Aires, Argentina; 4 Harran University, Şanlıurfa, Turkey; 5 Medical University of Vienna, Wien, Austria; 6 I. Department of Medicine, University Medical Center Hamburg-Eppendorf, Hamburg, Germany; 7 European Reference Network on Hepatological Diseases (ERN RARE-LIVER); 8 University Centre for Gastrointestinal and Liver Diseases, Basel, Switzerland; 9 IRCCS Humanitas Research Hospital Milan, Milan, Italy; 10 Maastricht University Medical Center, Maastricht, Netherlands; 11 Lithuanian University of Health Sciences, Kaunas, Lituania; 12 Donostia University Hospital, Donostia, Spain; 13 Servicios de Ap Digestivo y Farmacologia Clínica, Hospital Universitario Virgen de la Victoria, IBIMA, Universidad de Málaga, Malaga, Spain; 14 Hépatologie et Transplantation Hépatique, Hôpital Paul Brousse, Villejuif, France; 15 Städtisches Krankenhaus Kiel, Kiel, Germany; 16 Aarhus University Hospital, Aarhus, Denmark; 17 Ospedale San Raffaele, Milano, Italy; 18 Policlinico San Marco, Zingonia, Italy; 19 University Hospital RWTH Aachen, Aachen, Germany; 20 Hepatology, University Clinic for Visceral Surgery and Medicine, Inselspital Bern, University of Bern, Bern, Switzerland; 21 University of Rosario School of Medicine, Rosario, Argentina; 22 Centre Hospitalier Universitaire de Rennes, Rennes, France; 23 Istituti Clinici Pavia-Vigevano UO Epatologia Oncologica, Vigevano, Italy; 24 Department of Gastroenterology and Hepatology, Kurashiki Central Hospital, Kurashiki, Japan; 25 Department of Clinical Immunology and Allergy, Royal North Shore Hospital, St Leonards, Australia; 26 Department of Pathology, Akdeniz University Faculty of Medicine, Antalya, Turkey; 27 Department of Pathology, Inonu University Faculty of Medicine, Malatya 44280, Turkey; 28 Epatocentro Ticino, Lugano, Switzerland; 29 MowatLabs, Faculty of Life Sciences & Medicine, King’s College London, King’s College Hospital, London, UK; 30 Centro de Investigación Biomédica en Red Enfermedades Hepáticas y Digestivas (CIBERehd), Madrid, Spain; 31 Humanitas University, Pieve Emanuele, Milan, Italy; 32 Department of Gastroenterology and Hepatology, Graduate School of Medicine, Kyoto University, Kyoto, Japan

**Keywords:** acute liver injury, SARS-CoV-2 vaccines, liver histology, autoimmune liver serology, AIH, autoimmune hepatitis, ALP, alkaline phosphatase, ALT, alanine aminotransferase, AMA, anti-mitochondrial antibody, ANA, anti-nuclear antibody, AST, aspartate aminotransferase, COVID-19, coronavirus disease 2019, DILI, drug-induced liver injury, IAIHG, International Autoimmune Hepatitis Group, IFT, indirect immunofluorescence, LKM, liver kidney microsomal, LT, liver transplantation, PCA, parietal cell antigen, pIgG, polyreactive IgG, SARS-CoV-2, severe acute respiratory syndrome coronavirus type 2, SLA, soluble liver antigen, SMA, anti-smooth muscle antibody, ULN, upper limit of normal

## Abstract

**Background & Aims:**

Liver injury with autoimmune features after vaccination against severe acute respiratory syndrome coronavirus type 2 (SARS-CoV-2) is increasingly reported. We investigated a large international cohort of individuals with acute hepatitis arising after SARS-CoV-2 vaccination, focusing on histological and serological features.

**Methods:**

Individuals without known pre-existing liver diseases and transaminase levels ≥5x the upper limit of normal within 3 months after any anti-SARS-CoV-2 vaccine, and available liver biopsy were included. Fifty-nine patients were recruited; 35 females; median age 54 years. They were exposed to various combinations of mRNA, vectorial, inactivated and protein-based vaccines.

**Results:**

Liver histology showed predominantly lobular hepatitis in 45 (76%), predominantly portal hepatitis in 10 (17%), and other patterns in four (7%) cases; seven had fibrosis Ishak stage ≥3, associated with more severe interface hepatitis. Autoimmune serology, centrally tested in 31 cases, showed anti-antinuclear antibody in 23 (74%), anti-smooth muscle antibody in 19 (61%), anti-gastric parietal cells in eight (26%), anti-liver kidney microsomal antibody in four (13%), and anti-mitochondrial antibody in four (13%) cases. Ninety-one percent were treated with steroids ± azathioprine. Serum transaminase levels improved in all cases and were normal in 24/58 (41%) after 3 months, and in 30/46 (65%) after 6 months. One patient required liver transplantation. Of 15 patients re-exposed to SARS-CoV-2 vaccines, three relapsed.

**Conclusion:**

Acute liver injury arising after SARS-CoV-2 vaccination is frequently associated with lobular hepatitis and positive autoantibodies. Whether there is a causal relationship between liver damage and SARS-CoV-2 vaccines remains to be established. A close follow-up is warranted to assess the long-term outcomes of this condition.

**Impact and implications:**

Cases of liver injury after vaccination against severe acute respiratory syndrome coronavirus type 2 (SARS-CoV-2) have been published. We investigated a large international cohort of individuals with acute hepatitis after SARS-CoV-2 vaccination, focusing on liver biopsy findings and autoantibodies: liver biopsy frequently shows inflammation of the lobule, which is typical of recent injury, and autoantibodies are frequently positive. Whether there is a causal relationship between liver damage and SARS-CoV-2 vaccines remains to be established. Close follow-up is warranted to assess the long-term outcome of this condition.

## Highlights

• We report on 59 individuals with acute liver injury after SARS-CoV-2 vaccination.
• Histology shows mostly lobular hepatitis without relevant fibrosis.
• 65% were ANA positive, 54% were SMA positive, 35% had elevated IgG.
• Transaminases were normal in 65% of cases at 6 months, one patient required liver transplantation.

## Graphical abstract

**Figure:**
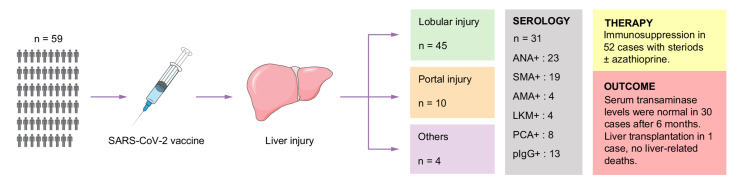

## Introduction

The ongoing pandemic caused by severe acute respiratory syndrome coronavirus 2 (SARS-CoV-2), a highly transmissible and pathogenic virus that causes coronavirus disease 2019 (COVID-19), has had a devastating global impact, which led to the unprecedentedly fast development of anti-COVID-19 vaccines. The vaccines are highly effective in preventing COVID-19, particularly in reducing the incidence of severe and fatal outcomes.^1^ Available vaccines have been developed using several different platforms, including mRNA vaccines, replication incompetent vector vaccines, inactivated vaccines and recombinant protein vaccines. While mild local and systemic side effects are relatively common, severe adverse reactions have been reported rarely, particularly anaphylaxis and myocarditis after mRNA vaccines, and immune thrombotic thrombocytopenia after viral vector vaccines.[2], [3], [4] In addition, the mRNA vaccines, which include BNT162b2 and mRNA-1273, can trigger the interferon pathway as part of their mechanism of action, raising some concerns regarding the possibility of vaccine-induced autoimmunity.^5^ However, according to a recent epidemiological study from Hong Kong, the incidence of severe autoimmune diseases did not increase after the start of the mass vaccination campaign.^6^

While registration trials did not detect liver injury as a side effect of SARS-CoV-2 vaccines, immunization of billions of people has led to the report of an increasing number of cases of acute hepatitis following vaccination.[7], [8], [9], [10], [11], [12], [13], [14], [15], [16], [17], [18], [19] According to a retrospective study carried out in the USA, the frequency of unexplained elevation of liver tests after SARS-CoV-2 vaccination is 0.038%, which is lower than the frequency after influenza vaccination.^17^

Case reports of acute hepatitis arising after SARS-CoV-2 vaccines often show positive autoantibodies, elevated IgG levels, interface hepatitis on liver histology and response to immunosuppressive treatment, raising the question as to whether this condition may be autoimmune hepatitis (AIH) triggered by vaccination.^7^^,^[10], [11], [12], [13], [14], [15]^,^^19^ A tertiary center in Germany, however, has not observed an increased incidence of AIH cases in 2021, after the introduction of the SARS-CoV-2 vaccines.^20^ Classical AIH is a rare chronic inflammatory liver condition characterized by female preponderance, high transaminase and serum IgG levels, positive autoantibodies, interface hepatitis on liver histology and a swift response to steroid treatment.^21^ As current knowledge mostly stems from case reports, little is known on key clinical, histological and immunological features of SARS-CoV-2 vaccine-associated liver injury. The aim of this international study was to collect clinical, pathological and serological data on a large number of individuals with acute liver injury diagnosed after a SARS-CoV-2 vaccination and review them centrally in order to define the key characteristics of this novel condition.

## Patients and methods

### Study population

Cases were collected from members of the International AIH Group (IAIHG) and the European Reference Network on Hepatological Diseases (ERN RARE-LIVER). Inclusion criteria were: elevation of transaminase levels ≥5x the upper limit of normal (ULN) occurring within 3 months from any vaccination against SARS-CoV-2 with available liver biopsy for central review and a clinical follow-up of at least 3 months or until liver transplantation (LT)/death, whichever came first, from diagnosis of acute liver injury. Exclusion criteria were: a known history of autoimmune liver disease (AIH; primary biliary cholangitis; primary sclerosing cholangitis); acute or chronic viral hepatitis including hepatitis A, B, C, D or E; history of LT.

All patients provided written informed consent. All procedures were conducted in accordance with the appropriate ethics committee.

### Definitions

Heterologous vaccination = exposure to a combination of vaccines (mRNA, vectorial, inactivated or protein-based).

Remission = alanine aminotransferase (ALT) normalization at 3 months after diagnosis.^22^
Relapse = any increase of transaminase levels after initial improvement.

The following variables were collected at diagnosis: sex; age at liver injury; date and name of each vaccine dose against SARS-CoV-2; date of hepatitis diagnosis; date of liver biopsy; re-exposure to a SARS-CoV-2 vaccine after the diagnosis of hepatitis; liver biochemistry and international normalized ratio (INR) at diagnosis, and 3 and 6 months after the diagnosis of hepatitis; concomitant autoimmune diseases; concomitant medications; medications for vaccine side effects; local autoantibody testing; treatment for hepatitis including name and dose of drug, date of treatment start and cessation. The liver injury pattern was categorized according to the R ratio value, defined as serum ALT/ULN divided by serum alkaline phosphatase (ALP)/ULN: R >5 defines a hepatocellular pattern of injury, R between 2 and 5 defines a mixed pattern, and R <2 defines a cholestatic pattern.^23^ The clinical severity of liver injury was assessed according to the original and revised Hy’s laws.^24^^,^^25^

### Histology

Liver biopsies were reviewed by an experienced histopathologist (YZ). Glass or digital slides were sent to the central reviewer, and inflammatory activity and fibrosis stages were assessed according to the modified Ishak’s scoring system.^26^ Only scarring fibrosis, but not collapsed stroma, was counted for fibrosis staging. Plasma cell or eosinophilic aggregates defined as the presence of ≥5 cells in a circular spot with a diameter of 150 μm were assessed. Finally, the recently proposed AIH pathological criteria were also applied, in addition to the simplified IAIHG criteria.^27^^,^^28^

### Autoantibodies

Serum of 31 patients was available for central testing. Autoantibody testing was performed after shipment of frozen serum samples at Hannover Medical School, Hannover/Germany. The presence of autoantibodies was tested in all serum samples via indirect immunofluorescence (IFT) on sections of frozen rodent liver, stomach and kidney (AESKUSLIDES, AESKU-Diagnostics) and on HEp2 cells (ZENIT-Autoimmunity Reagents, Menarini Diagnostics) as recommended by current guidelines,^29^ and via a liver line immunoassay (IMTEC-Leber-LIA, Human Gesellschaft für Biochemie und Diagnostika) including target antigens for anti-liver kidney microsomal (LKM) type 1 (CYP2D6), anti-mitochondrial (AMA) (pyruvate dehydrogenase-E2), anti-soluble liver antigen (SLA) (O-Phosphoseryl-TRNA(Sec) Selenium Transferase), anti-gp210 and anti-sp100 antibodies. Sera giving a LKM pattern were further investigated by western blots against CYP2D6, CYP2C9 and family 1 uridine 5’-diphosphate glucuronosyltransferase (LKM-1, 2 and 3, respectively). In case of positive staining of parietal cells on frozen stomach sections, samples were subsequently tested for the presence of IgG antibodies against parietal cell antigen (PCA) via a line immunoassay (Gastro-5-Line, Orgentec Diagnostika). We tested for the presence of polyreactive IgG (pIgG), reported to be elevated in untreated AIH and more specific and accurate to distinguish AIH from non-AIH liver diseases, via a custom-made ELISA containing BSA as a blocking reagent and huntingtin-interacting protein 1-related protein (HIP1R) as an autoantigen in a single 1:100 dilution as published recently.^30^

### Statistical analysis

Categorical variables are expressed as numbers and percentages; continuous variables are expressed as median and range. Statistical analysis was performed using SPSS Version 22.0. The Fisher’s exact test was used to compare categorical data between two groups. The Mann-Whitney *U* test was used to compare quantitative data between two groups. *P* values below 0.05 (two-tailed) were considered significant in all analyses.

## Results

### Clinical features

Patient characteristics at the time of the hepatitis diagnosis, treatment and outcomes are summarized in Table 1. Data on 11 patients were published before the centralized histological and serological evaluation presented here; their follow-up has been updated.^9^^,^^13^^,^[31], [32], [33]

**Table 1:** **Demographic and clinical data of individuals (n** = **59) with liver injury after vaccination against SARS-CoV-2**.

	n	%	Median (range)
Sex (female/male)	35/24	59/41	
Age			54 (19–92)
SARS-CoV-2 infection before liver injury	5	9	
**Vaccination**			
Heterologous vaccination	8	14	
Last vaccine before liver injury			
mRNA-1273 (Moderna)	12	20	
BNT162b2 (Pfizer)	30	51	
AZD1222 (AstraZeneca)	11	19	
Gam-COVID-Vac (Sputnik V)	5	9	
BBIBP-CorV (Sinopharm)	1	2	
Number of vaccinations before liver injury			
1	20	34	
2	37	63	
3	2	3	
Vaccine to hepatitis (days)			24 (1-74)
Medication for vaccine side effects			
No	33	56	
Yes	5	8	
Unknown	21	36	
**Laboratory values at diagnosis**			
ALT/ULN			24.0 (5.0–111.3)
AST/ULN			22.1 (3.0–169.1)
ALP/ULN			1.4 (0.5–8.2)
GGT/ULN			4.3 (0.4–39.0)
Total bilirubin/ULN (n = 56)			4.7 (0.4–34.4)
INR (n = 57)			1.2 (0.7–3.2)
IgG (g/L) (n = 58)			17.3 (6.6–39.9)
IgG >16 g/L	40	68	
IgM (g/l) (n = 48)			1.2 (0.2–9.8)
Original Hy’s law satisfied (n = 56)	32	57	
New Hy’s law satisfied (n = 56)	30	54	
Liver injury pattern R ratio (n = 58)			
Hepatocellular (R≥5)	55	95	
Mixed (R 2-5)	3	5	
**Histology**			
Centralized liver biopsy Ishak score			
Interface hepatitis			2 (0-4)
Confluent necrosis			2 (0-6)
Lobular hepatitis			3 (0-4)
Portal inflammation			2 (0-3)
Total necro-inflammatory activity			9 (0-14)
Fibrosis			1 (0-6)
Simplified IAIHG criteria			
Typical	14	24	
Compatible	34	58	
Atypical	11	19	
New histological criteria^27^			
Likely	41	70	
Possible	13	22	
Unlikely	5	8	
**Local autoantibody testing**			
ANA HEp2 cells, positive (n = 58)	43	74	
ANA titers HEp2 cells, ≥1:160	28/43	65	
ANA pattern HEp2 cells (n = 34)			
Homogeneous	16		
Fine speckled/speckled	13		
Nucleolar	4		
Mixed (homogeneous+speckled)	2		
Cytoplasmatic pattern HEp2 cells	3	5	
Reticular	2		
Granular	1		
SMA, positive (n = 59)	22	37	
SMA titer ≥1:160	12	55	
Anti-LKM, positive (n = 52)	4	8	
Anti-LKM titer, 1:160	4		
Anti-LC1, positive (n = 32)	0		
Anti-SLA, positive (n = 45)	0		
AMA positive (n = 55)	5	9	
AMA titer ≥1:160	4		
ANCA, positive (1:1,280) (n = 35)	1	3	
**Treatment of post-vaccine hepatitis**			
Immunosuppression (steroids ± azathioprine)	52	88	
Days from liver biopsy to treatment start			2 (-107 to 254)
Steroids therapy (prednisolone-equivalent)			50 mg/day (10-625)
Azathioprine addition to steroids	7	12	50 mg/day (25–150)
**Outcome**			
Spontaneous remission	5	9	
Relapse treated with steroids after spontaneous remission	2	3	
Remission with IS & successful IS withdrawal	10	17	
Remission with IS & IS withdrawal still ongoing	23	39	
Remission with IS & IS withdrawal failure	4	7	
Improvement but no remission despite IS treatment	12	20	
Liver transplantation	1	2	
Death, non-liver-related	3	5	

ALP, alkaline phosphatase; ALT, alanine aminotransferase; AMA, anti-mitochondrial antibody; ANA, anti-nuclear antibody; ANCA, anti-neutrophil cytoplasmic antibody; AST, aspartate aminotransferase; GGT, gamma-glutamyl-transferase; IAIHG, international autoimmune hepatitis group; INR, international normalized ratio; IS, immunosuppression; LKM, liver kidney microsomal; SLA, soluble liver antigen; SMA, anti-smooth muscle antibody; R ratio: ALT/ULN divided by ALP/ULN; SARS-CoV-2, severe acute respiratory coronavirus 2; ULN, upper limit of normal.

**Fig. 1:**
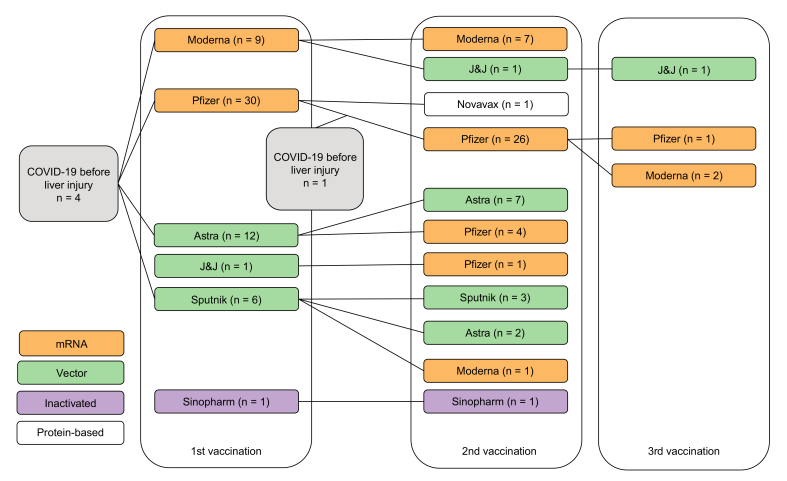
Sequence of SARS-CoV-2 vaccinations and infections before the onset of liver injury. COVID-19, coronavirus disease 2019; SARS-CoV-2, severe acute respiratory coronavirus 2.

Fifty-nine patients, from 26 centers in 11 countries were recruited according to the inclusion and exclusion criteria. The majority were female, median age at diagnosis of hepatitis was 54 years. Five had a history of COVID-19 before hepatitis (Fig. 1). Patients were exposed to seven different SARS-CoV-2 vaccines (mRNA-based vaccines: mRNA-1273 [Moderna] and BNT162b2 [BioNTech/Pfizer]; non-replicative virus vector vaccines: AZD1222 [AstraZeneca], Ad26.COV2.S [Johnson & Johnson] and Gam-COVID-Vac [Sputnik V]; vaccine with inactivated SARS-CoV-2: BBIBP-CorV [Sinopharm]; and protein-based vaccines: NVX-CoV2373 [Novavax]) in various combinations before the diagnosis of liver injury (Fig. 1). Hepatitis was diagnosed after the second vaccine dose in the majority of patients (Table 1). The median time from last vaccine dose to diagnosis of hepatitis was 24 days. Thirty-six patients (61%) were on other medications and/or had a history of other medications in the 12 weeks preceding the liver injury (Table S1); none was on steroids, while three were on immunosuppressants (azathioprine, anti-CD20, anti-IL23). Eighteen (31%) had an extrahepatic autoimmune comorbidity (Table S2). Five took medications to treat vaccine side effects, including acetaminophen at a dose of 1–1.5 g/day in all cases, and diclofenac in one case.

**Fig. 2:**
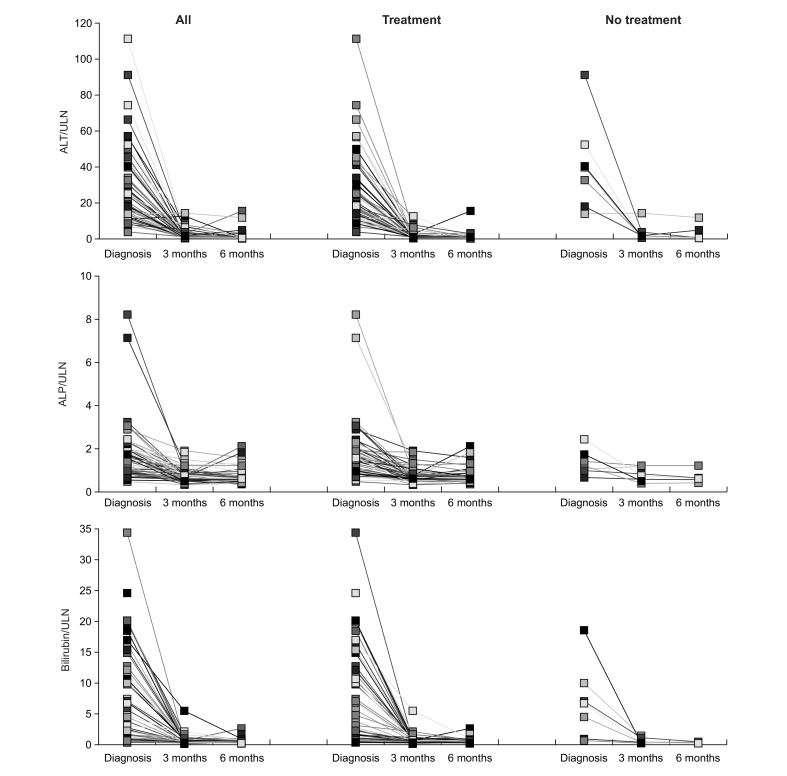
Longitudinal development of biochemistry tests in liver injuries associated with SARS-CoV-2 vaccines. ALP, alkaline phosphatase; ALT, alanine aminotransferase; SARS-CoV-2, severe acute respiratory coronavirus 2; ULN, upper limit of normal.

Laboratory test values obtained at presentation in the participating centers were normalized to the local ULN (Table 1 and Fig. 2). The liver enzyme pattern was hepatocellular in the vast majority of cases and mixed in a small minority; none had a cholestatic pattern.^23^ Total IgG was elevated (>16 g/L) in two-thirds of cases (Table 1). Acute liver failure including hepatic encephalopathy manifested in a single patient, the only one to require LT (113 days after re-exposure to BNT162b2 vaccine).

### Histology

Centralized liver biopsy Ishak’s score is summarized in Table 1.

**Fig. 3:**
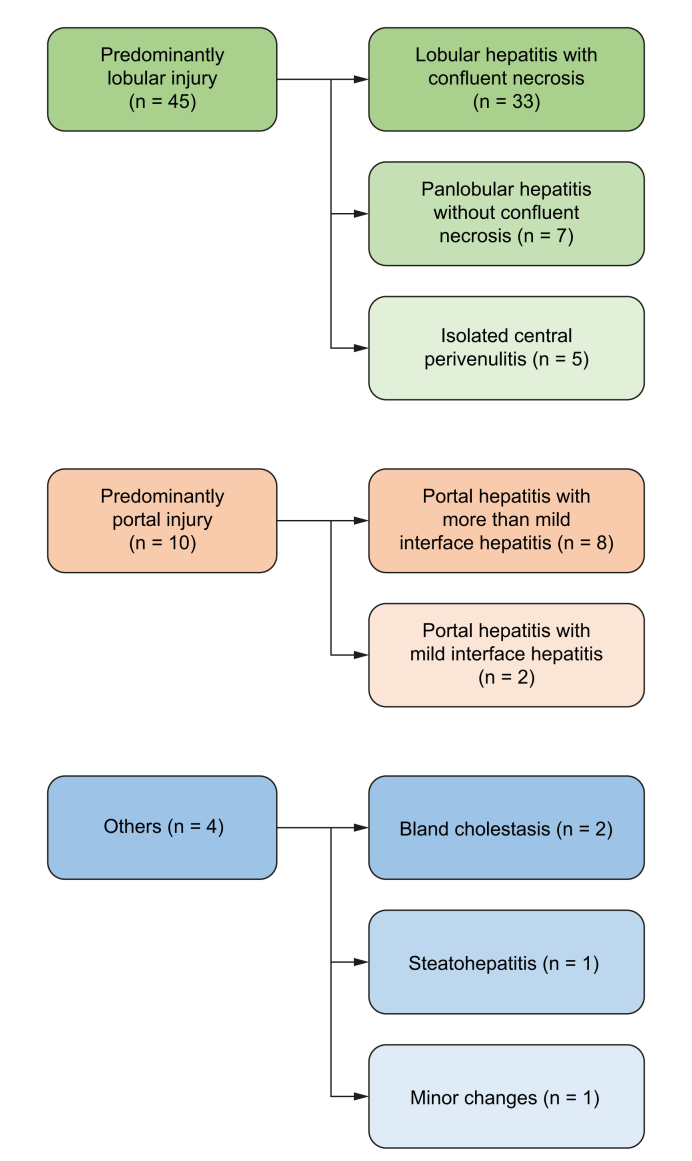
Histology of liver injury associated with SARS-CoV-2. The histological injury pattern can be categorized into predominant lobular (upper column) as well as predominant portal (mid column) injury pattern, each with subclassifications, and a small group of different manifestations (lower column). SARS-CoV-2, severe acute respiratory coronavirus 2.

**Fig. 4:**
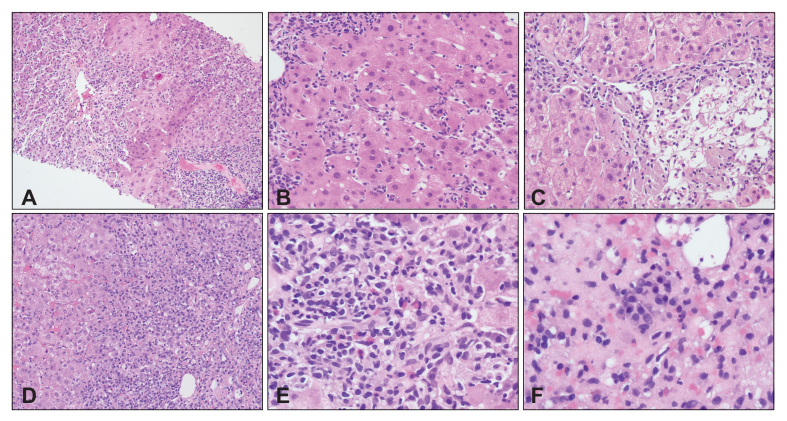
Representative histological pattern of liver injury associated with SARS-CoV-2. Outlined are representative examples for the histological types from Fig. 3: (A) Lobular hepatitis with confluent necrosis (100x); (B) Panlobular hepatitis (200x); (C) Isolated central perivenulitis; (D) Portal hepatitis with interface injury (100x); (E) Eosinophilic aggregates (400x); (F) Plasma cell aggregates (400x).

According to the predominant pattern of injury, the cases were classified into the following categories (Fig. 3, Fig. 4):

Predominantly lobular injury (n = 45, 76%): lymphocytic infiltration, focal necrosis and acidophilic bodies were observed in the parenchyma. Although most cases had portal inflammation, the necro-inflammatory changes were more conspicuous within the parenchyma than in the portal tracts. Variable degrees of confluent necrosis ranging from perivenular zonal necrosis to multiacinar parenchymal loss were identified in 33 cases, while seven cases showed features of panlobular hepatitis without confluent cell loss. The remaining five cases had a selective perivenular injury with confluent cell loss, in keeping with isolated central perivenulitis. Five cases among this group had conspicuous lobular cholestasis (cholestatic hepatitis). Another case also demonstrated multiple, small epithelioid granulomas in the parenchyma. None had cholangiopathic changes.

Predominantly portal injury (n = 10, 17%): portal-based lymphocytic infiltration was observed. Most cases had lobular inflammation and/or confluent necrosis, but the degree of inflammation was higher in portal tracts than in the parenchyma. Two cases had mild interface hepatitis, while eight cases showed more than mild interface injuries. None had cholangiopathic changes. Fibrosis stage was periportal fibrosis in six cases (Ishak’s stage 1-2), early bridging fibrosis in three cases (stage 3) and cirrhosis in one case (stage 6).

Table 2 compares microscopic features between the two groups. As expected, the degrees of interface hepatitis and portal inflammation were higher in cases of predominantly portal injury, while the degrees of lobular inflammation and confluent necrosis were higher in cases of predominantly lobular injury. Fibrosis was more advanced in cases of portal injury than in those of lobular injury. Plasma cell aggregates were common in both groups (62-80%), and eosinophil aggregates were also seen in 40-50% of cases. According to the newly proposed AIH histopathology criteria, 70-80% of cases were classified as likely AIH^27^ (Table 1).

**Table 2:** **Histological comparison between cases of predominantly lobular or portal hepatitis**.

	Lobular hepatitis	Portal hepatitis	*p* value
	(n = 45)	(n = 10)	
Interface hepatitis			
0-1	23 (51%)	2 (20%)	0.041
2	13 (29%)	4 (40%)	
3	9 (20%)	2 (20%)	
4	0	2 (20%)	
Confluent necrosis			
0	7 (16%)	7 (70%)	0.009
1-2	12 (26%)	2 (20%)	
3-4	17 (38%)	1 (10%)	
5-6	9 (20%)	0	
Lobular necroinflammation			
0-1	2 (4%)	2 (20%)	0.004
2	13 (29%)	6 (60%)	
3	16 (36%)	2 (20%)	
4	14 (31%)	0	
Portal inflammation			
0-1	13 (29%)	0	0.005
2	26 (58%)	5 (50%)	
3	6 (13%)	5 (50%)	
4	0	0	
Fibrosis			
0	6 (13%)	0	0.006
1-2	36 (80%)	6 (60%)	
3-4	3 (7%)	3 (30%)	
5-6	0	1 (10%)	
Inflammatory cells			
Plasma cell aggregate	28 (62%)	8 (80%)	0.285
Eosinophil aggregate	18 (40%)	5 (50%)	0.562
Histological criteria			
Likely	32 (71%)	8 (80%)	0.572
Possible	13 (29%)	2 (20%)	
Unlikely	0	0	

Histological parameters were compared between cases of lobular and portal hepatitis using the Mann-Whitney *U* test. A probability of *p* <0.05 was considered to be significant.

Other patterns of injury (n = 4, 7%): Two cases showed bland cholestasis with bile casts in the canaliculi or the cytoplasm of hepatocytes, not associated with significant necro-inflammatory changes. One case showed features of active steatohepatitis. The last case had only minor microscopic changes.

Clinical features of cases with predominantly lobular or predominantly portal hepatitis were similar, except for higher aspartate aminotransferase (AST) and bilirubin at diagnosis in the first group (Table S3). The post-vaccination biopsy of the patient who progressed to LT showed lobular hepatitis with confluent necrosis and eosinophils. At LT, his liver showed post-necrotic stromal collapse, nodular transformation of the residual parenchyma and mild persistent lobular hepatitis.

### Autoantibodies

#### Local testing

A broad variety of autoantibody assays including IFT on rodent tissue sections and/or HEp2 cells as well as ELISAs were used in the participating centers, leading to inhomogeneous results that were difficult to compare (Table 1). Nuclear IFT on HEp2 cells was positive in most tested patients, being ≥1:160 in two-thirds, the staining pattern being mostly homogeneous or fine speckled/speckled (Table 1). Anti-smooth muscle antibody (SMA) was detected in one-third of the tested patients, with titers ≥1:160 in half of them. The IFT pattern on kidney tissue was not available. LKM, tested in 52 cases, was detected in four with a titer of 1:160 in all (Table 1). Anti-liver cytosol antibody was negative in all 32 patients tested. SLA was negative in all 45 tested cases. AMA was detected by IFT in five patients, four of whom had a titer ≥1:160. In one case, AMA, not tested by IFT, was positive on molecular testing.

#### Centralized testing (Table 3)

Serum samples for centralized and standardized autoantibody testing were available for 31/59 patients (52%) (Table 3). Two patients were negative for all tested specificities. ANA, tested by IFT on triple tissue, was present in three-quarters, most of whom had titers >1:160. ANA by HEp2 cells was positive in 27 (87%), with mostly a fine speckled staining pattern. SMA was present in 19 patients, with titers ≥1:160 in 11, half having an isolated vessel pattern and one-third having a vessel, glomerulus and tubule pattern on kidney sections. AMA was present in four patients by IFT (titers ≥1:160 in all), confirmed by molecular assay in all. LKM was found in four patients with moderate to high titers (1:80-1:160) in IFT, but without specificity for LKM-1, 2 or 3 by Western blot; one was positive for LKM-1 by line immunoassay. PCA was positive in eight cases. Twenty patients had more than one autoantibody. Polyreactive IgG with reactivity against BSA/HIP1R was detected in almost half of the patients.^30^

**Table 3:** **Centralized autoantibody testing in 31 individuals with liver injury after vaccination against SARS-CoV-2**.

	n	%
ANA on triple tissue, positive	23	74
ANA titers on triple tissue		
80	4	17
≥160	19	56
ANA on HEp2cells, positive	27	87
ANA titers on HEp2 cells		
80	6	22
≥160	21	68
ANA patterns on HEp2 cells		
Homogeneous	4	15
Fine speckled	15	56
Nucleolar	4	15
Centromere	1	4
Mixed patterns	3	11
SMA, positive	19	61
SMA titer		
80	8	42
≥160	11	35
SMA pattern		
V	10	53
VGT	7	37
AMA, positive	4	13
AMA titer, ≥160	4	
LKM, positive	4	13
LKM titer		
80	1	
160	3	
Western blot, positive for LKM1, LKM2, LKM3	0	
PCA, positive	8	26
PCA titer		
80	2	
≥160	6	
Liver LIA, positive	5	16
Liver LIA		
LKM1	1	
AMA M2	4	
pIgG (cut-off = 1,27), positive	13	46.4
pIgG (normalized arbitrary units), median (range)	1.2 (0.7–2.7)	

AMA, anti-mitochondrial antibody; ANA, anti-nuclear antibody; LKM, liver kidney microsomal; PCA, parietal cell antibody; pIgG, polyreactive IgG; SARS-CoV-2, severe acute respiratory coronavirus 2; SLA, soluble liver antigen; SMA, anti-smooth muscle antibody; V, vessel; VGT, vessel, glomerulus; tubule.

### Treatment and outcome

The treatment decision was made at each center according to local standards without a unified protocol. Most patients received immunosuppression (Table 1). Two patients were treated after 5 and 7 months from initial diagnosis because of relapse after spontaneous remission. Therapy was initiated with steroids in all cases with a median equivalent dose of 50 mg prednisolone per day (range: 10-625 mg/day); four patients received ≥100 mg prednisolone-equivalent/day intravenously due to severe presentation with high bilirubin (>10x ULN). Nine patients were started on treatment before undergoing liver biopsy, at a median time of 28 days, range 1-107. Various treatment schedules were used, including prednisone (n = 18), prednisolone (n = 16), meprednisone (n = 8), methylprednisolone (n = 5), prednisolone + azathioprine (n = 6), and budesonide (6 mg/day) + azathioprine (n = 1).

Liver tests improved after 3 months in all patients (Fig. 2). There were no significant differences between treated and untreated participants in terms of demographics and clinical characteristics, vaccine type, time from vaccination to liver injury, histological and serological features, and outcome. ALT at 3 months after the onset of liver injury was normal in 24/58 patients (one patient died of cardiac decompensation 2 months after the onset of liver injury); 6-month data, available for 46 patients (80%), showed normal ALT levels in 30 patients (64%), of whom 23 were still on treatment. The three patients on long-term immunosuppression before vaccination were treated with steroids; two are still on treatment without complete ALT normalization after 3 months, and one could discontinue steroids after 5 months without relapse. At submission of this manuscript, 14/59 (24%) patients were in remission without immunosuppression (five after spontaneous remission, nine after successful immunosuppression withdrawal), 23/59 (39%) were in remission during immunosuppression withdrawal, 12/59 (20%) experienced a decrease of transaminase levels without normalization on immunosuppression, 6/59 (10%) underwent a relapse after remission (two after spontaneous remission, four during immunosuppression weaning). One patient needed a LT, three (5%) died of non-liver related causes, of whom one died of cardiac decompensation, and one, who had undergone remission and successful immunosuppression withdrawal, of progression of pre-existing extrahepatic cancer; a 77-year-old woman, without pre-existing conditions, died of pulmonal and cerebral aspergillosis while on immunosuppressive treatment; the initial prednisone dose was 60 mg/day. All patients with fatal outcome or requiring LT were initially treated. The only patient with established cirrhosis responded well to steroid treatment but relapsed after treatment discontinuation.

### Subgroup analyses

The total cohort was heterogeneous in several aspects including co-medication, type of SARS-CoV-2 vaccine, time point of diagnosis of liver injury in the vaccination sequence, and presence of advanced liver fibrosis.

#### Time of liver injury

Patients in whom liver injury was diagnosed after the first vaccination exhibited less severe ALT elevation (median 17.8 ULN *vs.* 26.5 ULN, *p* = 0.012) but higher IgG levels (median 19.0 g/l *vs.* 16.5 g/l, *p* = 0.026), with a higher frequency of SMA positivity at central testing (8/8 *vs.* 12/23, *p* = 0.015) compared to patients in whom the liver injury manifested after a second or third vaccination. Twenty patients presented with acute liver injury after the first vaccine dose, of whom 10 received a vectorial vaccine; in contrast, out of the 39 patients presenting with acute liver injury after the second or third vaccine dose, only six received a vectorial vaccine (*p* = 0.006). Although patients with liver injury after the first or the second/third vaccination were treated with the same frequency and with comparable initial steroid doses and had comparable outcome at 6 months, AST levels were slightly higher at 3 months (1.4 ULN *vs.* 0.9 ULN, *p* = 0.051) and bilirubin was slightly higher (0.6 ULN *vs.* 0.4 ULN, *p* = 0.07) at 6 months follow-up in those who develop liver injury after the first dose.

#### Type of vaccine

Patients with liver injury after mRNA vaccines had higher transaminase levels (ALT 26.2x ULN *vs.* 14.0x ULN, *p* = 0.003; AST 25.1x ULN *vs.* 11.2x ULN, *p* = 0.008) and higher impairment of coagulation (INR 1.3 *vs.* 1.1, *p* = 0.012) than those who developed hepatitis after vector vaccines. Treatment and treatment response were similar in both groups. A comparison between the two mRNA vaccines was limited by the small patient numbers. Nonetheless, patients with hepatitis after mRNA-1273 (n = 12) had more severe histological injury (Ishak necroinflammation grade 11 *vs.* 9, *p* = 0.001), higher SMA titers (≥1:160 in 8/8 *vs.* in 2/8, *p* = 0.007) and higher pIgG concentrations (median 1.6 *vs.* 0.9, *p* = 0.012) than after BNT162b2 (n = 30). The comparison between the two vector vaccines AZD1222 (n = 11) and Gam-COVID-Vac (n = 5) did not show differences between these two small cohorts (data not shown).

#### Advanced liver fibrosis

The absence of advanced liver fibrosis in the work-up of an acute liver injury suggests drug-induced liver injury (DILI) or AIH-like DILI as more probable than AIH.^25^ Therefore, the liver histological injuries after SARS-CoV-2 vaccines were compared regarding the absence (n = 52) or presence (n = 7) of advanced liver fibrosis (defined as ≥Ishak F3). F3 was used as a threshold as portal expansion in acute hepatitis is potentially interpreted as F1 or F2. There were no significant differences in the liver enzyme elevation, IgG levels, bilirubin, or INR at presentation (Table S4). Treatment, liver function tests at 3 and 6 months and the overall comparison of the outcome was not significantly different between the two fibrosis groups. However, this comparison is limited by the small number of patients with advanced fibrosis.

### Re-challenge

Fifteen patients were re-exposed to a SARS-CoV-2 vaccine after the diagnosis of hepatitis (Table 4).

**Table 4:** **Re-challenge**.

Patient ID	Sex	Age	Time from hepatitis to re-exposure (days)	Vaccine(s) prior to hepatitis	Vaccine(s) after hepatitis	Immunosuppression at time of re-exposure	Outcome after re-exposure
**Homologous vaccination**							

9	F	67	195	Gam-COVID-Vac (sputnik adenovirus)	ChAdOx1 (astrazeneca adenovirus)	Yes (prednisone 6mg/d + azathioprine 75mg/d)	No relapse
11	F	63	134	Gam-COVID-Vac	ChAdOx1	Yes (meprednisone)	No relapse
16	F	46	60	ChAdOx1	ChAdOx1	Yes (prednisone)	No relapse
17	M	72	55	ChAdOx1	ChAdOx1	Yes (prednisone)	No relapse
2	M	51	34	mRNA-1273	mRNA-1273	No	No relapse
12	F	75	128	Gam-COVID-Vac	Gam-COVID-Vac	Yes (prednisone 25mg/d)	Re-exposure while still high transaminases without worsening
18	M	53	25	BNT162b2	BNT162b2	Yes (prednisone 16mg/d)	Relapse, finally requiring liver transplantation
20	F	61	16	ChAdOx1	ChAdOx1	No	Relapse, treated successfully with steroids (no treatment of first episode)
7	M	78	2	mRNA-1273	mRNA-1273	No	Relapse, treated successfully with steroids (no treatment of first episode) (Ad26.COV2.S 6 months later without relapse on prednisone 5mg/d)
19	M	62	21	BNT162b2	BNT162b2	No	Re-exposure while still high transaminases without steroids, improvement but no remission on prednisone+azathioprine

**Heterologous vaccination**							

17	M	63	181	mRNA-1273	Ad26.COV2.S (adenovirus) (2 doses 4 months apart)	Yes (prednisone 5mg/d)	No relapse
13	F	68	99	ChAdOx1	BNT162b2	Yes (prednisone and azathioprine)	No relapse
57	F	52	426	BNT162b2	NVX-CoV2373 (recombinant)	Yes (prednisone 5mg/d)	No relapse
14	M	33	92	ChAdOx1	BNT162b2	No	No relapse
15	F	58	66	ChAdOx1	BNT162b2	No	Re-exposure while still high transaminases, treated successfully with steroids

Ten received the same vaccine class, of whom six had no relapse (five on and one off immunosuppression), three relapsed (one on and two off immunosuppression), and one was re-vaccinated while transaminase levels were still elevated and showed improvement upon subsequent corticosteroid treatment. The patient who relapsed despite treatment finally needed a LT.

Five patients were re-exposed to a different vaccine class (heterologous vaccination), of whom four had no relapse (three on and one off immunosuppression), and one, off immunosuppression at the time of re-vaccination, was re-exposed while transaminases were still elevated and responded well to steroids.

When patients were grouped according to re-vaccination with or without ongoing immunosuppression, of those with ongoing immunosuppression, 8/10 had no relapse or worsening of liver injury upon re-vaccination, one relapsed and one was vaccinated while elevated liver enzymes were still present. Of those without immunosuppression, 3/6 had no relapse, 1/6 had a relapse and 2/6 were re-vaccinated while elevated liver enzymes were still present.

In summary, only one patient, who was rechallenged with the BNT162b2 vaccine, relapsed on immunosuppression, finally requiring a LT, and none of the four patients who had been rechallenged with heterologous vaccination while in remission (three on low-dose immunosuppression) relapsed.

## Discussion

To date, most reports of acute liver injury diagnosed after SARS-CoV-2 vaccination refer to single cases or small patient cohorts, except for a recent multicenter study by Efe *et al.*, which aimed to assess the clinical characteristics and outcome of hepatitis occurring after SARS-CoV-2 vaccination, irrespective of a previous history of liver disease.^31^ The present study focuses on centralized rigorous liver histology evaluation of patients without pre-existing liver conditions, to evaluate whether liver injury post-SARS-CoV-2 vaccination has specific features and can be distinguished from other types of acute onset hepatitis.

Liver histology showed a picture of predominant lobular hepatitis in three-quarters of cases, while predominant portal hepatitis was present in fewer than one-fifth of patients, supporting an acute onset of liver injury. Almost all patients in the present cohort were seropositive locally and at centralized testing for autoantibodies associated with AIH, frequently at high titers, and had high IgG, collectively suggesting a diagnosis of AIH or AIH-like DILI.^34^ The fact that only a few of them had advanced liver fibrosis, would support that elevation of transaminase levels following SARS-CoV-2 vaccination reflects acute liver injury in the absence of pre-existing unrecognized chronic liver damage, and therefore would favor AIH-like DILI. For those patients with established fibrosis, SARS-CoV-2 vaccination may have unmasked pre-existing undiagnosed chronic liver disease, including AIH. Of note, the majority of patients presented after the second vaccine dose, suggesting that repeated exposure increases the risk of liver injury with autoimmune features, an observation also reported in DILI.^35^ Some patients might have had subclinical liver inflammation after the first dose, which may explain the presence of liver fibrosis despite clinically acute presentation.

Criteria for differentiating classical AIH from AIH-like DILI are a matter of ongoing discussion: while AIH is characterized by long-term immunosuppression dependency and frequent presence of advanced fibrosis at diagnosis, the latter is characterized by a low relapse rate after withdrawal of a short-term steroid course.^25^ Ninety-two percent of our patients were treated with steroids, with or without azathioprine, and showed an excellent response, liver enzymes improving in all cases and normalizing in two-thirds after 6 months; however, as most of them are still on immunosuppression and the cohort follow-up is too short, it is impossible to determine whether they suffer from AIH-like DILI or classical AIH purely based on their response to treatment. In an attempt to evaluate whether published scoring systems could help in the differential diagnosis, we have applied both the simplified IAIHG diagnostic scoring system^28^ and the newly proposed ERN histological criteria.^27^ Neither provided helpful information, as 82% of patients scored as ‘typical or ‘probable’ AIH in the IAIHG diagnostic system and 92% of patients as ‘likely’ or ‘possible’ AIH in the ERN histology system. In particular, the new ERN histological criteria, which include the acute presentation of AIH characterized by lobular hepatitis, led to a more frequent rate of AIH likelihood (“likely”, 70%) compared to the IAIHG criteria (“typical”, 24%).^36^ This observation confirms that the diagnosis of AIH and the differential diagnosis with AIH-like DILI cannot be based solely on histology but requires a collegial approach to the clinical and laboratory findings. To evaluate the differential diagnostic role of pIgG, a new serological marker for AIH with a reported higher specificity and accuracy than conventional autoantibodies,^30^ we have tested our cohort and found pIgG less frequently than ANA and SMA. The follow-up of our cases and future studies are necessary to establish whether pIgG has a role in distinguishing AIH-like DILI from classical AIH with an acute presentation.

The question as to whether vaccines can trigger autoimmunity predates the COVID-19 pandemic. A meta-analysis addressing this question published in early 2020 could not find an increased incidence of autoimmune diseases in vaccinated people.^37^ Likewise, the current notion is that vaccines are not associated with flares of autoimmune diseases: in contrast, they rather prevent flares caused by vaccine-preventable infections.^38^ Nonetheless, *de novo* manifestations or aggravations of a variety of autoimmune diseases have been reported after different SARS-CoV-2 vaccinations.^39^

The overall similar phenotype of hepatitis occurring after mRNA or vectorial vaccines observed in this study may suggest that the liver injury is related to the spike protein antigen itself, rather than to a non-antigen specific immune-mediated damage, as reported by a recent case study.^40^

Most patients in the present cohort responded well to steroid-based therapy, though it is unknown how many would have improved spontaneously. Only one patient required LT for severe deterioration of liver injury after re-exposure to the same SARS-CoV-2 vaccine. It is of interest that of the 15 patients in our cohort who were re-exposed to vaccination, only three relapsed, all re-vaccinated with the same vaccine type.

Liver disease was not the cause of death of the three patients who died, but it is of concern that one of them had invasive pulmonary and cerebral aspergillosis in association with steroid treatment. As some two-thirds of patients in biochemical remission are still under immunosuppressive therapy at 6 months, longer follow-up is warranted to evaluate whether immunosuppression has indeed a beneficial effect on the prognosis of liver injury associated with SARS-CoV-2 vaccination.

The study of the pathogenic mechanisms in autoimmune disease is hampered by the inability to assess early events. The appearance of a liver autoimmune profile compatible with AIH shortly after the administration of an anti-SARS-CoV-2 vaccine offers the opportunity to investigate the events leading to liver autoimmunity by investigating its early stages, including virus/self cross-reactivity, maturation of the autoimmune response, and epitope spreading both at the T- and B-cell level.

The limitations of this multicenter study include reporting and selection bias. Participants were recruited mostly via networks focused on autoimmune as well as rare liver diseases and the contributing centers were mostly tertiary referral centers. The inclusion criteria of a liver biopsy might have caused a bias towards more severe and not rapidly self-limiting liver injuries. This is highlighted by the high treatment rate of 92% in the current study compared to a recently published multicenter cohort, in which only half of the patients had a liver biopsy and received immunosuppressive therapy.^31^ Both studies are large cooperative efforts, demonstrating that the condition is rare.

Our study gives no estimate of the incidence of liver injury after SARS-CoV-2 vaccination. When a high proportion of the world population is vaccinated within a few months, rare diseases – including AIH or AIH-like DILI due to other drugs – will be diagnosed close to a SARS-CoV-2 vaccination just by chance without any causal relationship. In contrast, flares or new onset of autoimmune diseases not associated with SARS-CoV-2 vaccines may not be recognized and reported with the same attention. Moreover, the occurrence of liver disease during the coronavirus pandemic can be underestimated owing to the overload of healthcare systems and to patient restraint in seeking medical attention. This might explain the reduced rate of newly diagnosed AIH during the pandemic in a tertiary referral center in Germany.^20^

The present study cannot prove or refute a causal relationship between SARS-CoV-2 vaccines and liver injury with autoimmune features. As most patients received other drugs during the 3 months preceding liver injury, other DILI triggers cannot be excluded.

In conclusion, acute liver injury with autoimmune features that shows a temporal association with SARS-CoV-2 vaccination is likely to be a heterogeneous condition requiring a thorough work-up and careful follow-up. Patients are often treated with immunosuppression, with a good short-term response, though firm indications on when to start immunosuppression are needed, to avoid adverse effects. This study does not justify withholding SARS-CoV-2 vaccination, which has prevented severe COVID-19 disease and death in millions of people.

## Financial support

The authors received no financial support to produce this manuscript.

## Authors’ contributions

All authors made substantial contributions to the conceptualization and design, the analysis, and data interpretation. BTBP and RT: conceptualization and supervision of the study, data interpretation, drafting of the manuscript, review and editing of the manuscript. GC, TK: data collection, data interpretation and statistical analysis. YZ: review of histological slides. AC, HW, DV, GMV, MIL, RJA: critical revision of the manuscript. BE, AMV, CE, AFS, JPW, MS, CB, AL, TJG, LK, AC, JP, EDM, IB, TDS, FP, FI, PDP, TB, MK, NS, FB, BG, GP, MU, HJ, GÖE, NKS: data collection. All authors have approved the final version of the manuscript.

## Data availability statement

Raw data are available from the corresponding author upon reasonable request.

## Conflict of interest

The authors do not have any conflict of interest pertaining to this study.

Please refer to the accompanying ICMJE disclosure forms for further details.
