# Supplementary material for: Histological and serological features of acute liver injury after SARS-CoV-2 vaccination
Source: JHEP Rep. 2022 Oct 13;5(1):100605. doi: 10.1016/j.jhepr.2022.100605 (PMC9691430; doi:10.1016/j.jhepr.2022.100605)
Supplement: Multimedia component 1 [file mmc1.pdf]

Histological and serological features of acute liver injury after SARS-CoV-2 vaccination

Greta Codoni, Theresa Kirchner, Bastian Engel, Alejandra Maria Villamil, Cumali Efe, Albert Friedrich Stättermayer, Jan Philipp Weltzsch, Marcial Sebode, Christine Bernsmeier, Ana Lleo, Tom JG Gevers, Limas Kupčinskas, Agustin Castiella, Jose Pinazo, Eleonora De Martin, Ingrid Bobis, Thomas Damgaard Sandahl, Federica Pedica, Federica Invernizzi, Paolo Del Poggio, Tony Bruns, Mirjam Kolev, Nasser Semmo, Fernando Bessone , Baptiste Giguet , Guido Poggi, Masayuki Ueno, Helena Jang, Gülsüm Özlem Elpek, Neşe Karadağ Soylu, Andreas Cerny, Heiner Wedemeyer, Diego Vergani, Giorgina Mieli-Vergani, M. Isabel Lucena, Raul J Andrade, Yoh Zen, Richard Taubert, Benedetta Terziroli Beretta-Piccoli

Table of contents

Table S1 2

Table S2 3

Table S3 4

Table S4 6

Table S1: Co-medications

Co-medications (n=59)	n
**Yes**	36 (61%)
**No**	23 (39%)
Anti-hypertensive drugs	21
Anti-hyperlipidemic drugs	10
Anti-diabetic drugs	9
Vitamins	8
Aminosalicylates	8
Proton pump inhibitors	7
Diuretics	7
L-thyroxine	5
Non-steroidal anti-inflammatory drugs	4
Anti-epileptic drugs	4
Monoclonal antibodies*	3
Herbal remedies	3
Selective serotonin re-uptake inhibitors	2
Anxiolitics/hypnotics	2
Anti-tumor drugs**	2
Azathioprine	1
Others	8

* Denosumab (RANKL-inhibitor); Ocrelizumab (anti-CD20); Risankizumab (anti-IL23)
** Letrozole, Ribociclib

Table S2: Autoimmune extrahepatic co-morbidities

**Autoimmune co-morbidities (n=59)**	**n**
**Yes**	18 (31%)
**No**	41 (69%)
Autoimmune thyroid disease	6
Psoriasis	2
Celiac disease	3
Sarcoidosis	1
Multiple sclerosis	2
Autoimmune hemolityc anemia	1
Ulcerative colitis	1
Crohn's disease	1
Rheumatoid arthritis	1
Cushing syndrome	1

Table S3: Comparison of clinical features between cases with predominantly lobular and predominantly portal hepatitis

	Lobular hepatitis N=45	Portal hepatitis N=10	p value	test
Female sex	28 (62%)	5 (50%)	0.49	Fisher's exact
Median age (years)	54	59	0.23	Mann-Whitney U
Last vaccine before hepatitis: mRNA	34 (75%)	6 (60%)	0.43	Fisher's exact
Heterologous vaccination	6 (13%)	1 (10%)	1.00	Fisher's exact
Hepatitis after first dose	19 (42%)	2 (20%)	0.30	Fisher's exact
Hepatitis after second dose	25 (55%)	7 (70%)	0.49	Fisher's exact
Hepatitis after third dose	1 (2%)	1 (10%)	0.33	Fisher's exact
Time from last vaccine to hepatitis diagnosis, median (days)	24	10	0.06	Mann-Whitney U
ALT at diagnosis, x ULN (median, U/l)	26 (1002)	15 (656)	0.27	Mann-Whitney U
AST at diagnosis, x ULN (median, U/l)	28 (968)	14 (469)	0.02	Mann-Whitney U
Alkaline phosphatase at diagnosis, x ULN (median, U/l)	171	121 (n=9)	0.09	Mann-Whitney U
Total bilirubin at diagnosis, x ULN (median, μmol/l)	138	29 (n=9)	0.03	Mann-Whitney U
Hepatocellular liver injury pattern	43	9 (n=9)	1.00	Fisher's exact
Mixed liver injury pattern	2	0	n.a.	Fisher's exact
Hy's criteria met	25	4 (n=9)	1.00	Fisher's exact
New Hy's law met	24	4 (n=9)	1.00	Fisher's exact
No concomitant medication	19	4	1.00	Fisher's exact
No medications for vaccine side effects	16 (n=26)	7 (n=7)	0.07	Fisher's exact
ANA positive	35 (n=44)	7 (n=9)	1.00	Fisher's exact
SMA positive	18	4 (n=7)	0.43	Fisher's exact
Anti-LKM positive	4 (n=38)	0 (n=8)	1.00	Fisher's exact
AMA positive (1 borderline)	6 (n=40)	0 (n=7)	0.57	Fisher's exact
IgG, g/l, (median)	16.9	18.8	0.78	Mann-Whitney U
IgM, g/l (median)	1.21(n=37)	1.61 (n=8)	0.40	Mann-Whitney U
Treatment	41	9	1.00	Fisher's exact
Normal ALT at 3 months	18 (n=42)	3 (n=7)	1.00	Fisher's exact

Normal ALT at 6 months	22 (n=29)	4 (n=7)	0.37	Fisher's exact

ALT, alanine aminotransferase; AST, aspartate aminotransferase; IgG/M Immunoglobulin, G/M; ULN, upper limit of normal; SMA, anti-smooth muscle antibody; ANA, anti-nuclear antibody; LKM, liver kidney microsomal; AMA, anti-mitochondrial antibody; n.a. not applicable.

Numbers shown in paranthesis as "n= " represent the number of patients for whom the piece of data is available.

Table S4: Comparison of clinical features between cases with and without advanced liver fibrosis

n=59		Ishak F0/1/2 (n=52)			Ishak F ≥3 (n=7)			p-value	test
		n	%	median (min; max)	n	%	median (min; max)		
**Sex**	male	21	40		3	43		.901	Chi²
**Age (years)**				54 (19;78)			60 (37;92)	.327	Mann-Whitney U
**COVID-19 before liver injury**	yes	4	8		1	14		.557	Chi²
**Vaccination**									
**Heterologous vaccination**	yes	7	14		1	20		.732	Chi²
**Last vaccine before hepatitis**	RNA	35	67		7	100		.200	Chi²
	vector	16	31		0	0			
	inactivated	1	2		0	0			
**Vaccine before hepatitis**	mRNA-1273 (Moderna)	10	19		2	29		.523	Chi²
	BNT162b2 (Pfizer)	25	48		5	71			
	AZD1222 (Astra)	11	21		0	0			
	Ad26.COV2.S (Johnson&Johnson)	0	0		0	0			
	Gam-COVID-Vac (Sputnik V)	5	10		0	0			
	BBIBP-CorV (Sinopharm)	1	2		0	0			

**Vaccine to hepatitis (days)**				24 (1;74)			19 (1; 48)	.573	Mann-Whitney U
**Hepatitis post vacc 1 vs 2/3**	post 1st vaccination	18	35		2	29		.751	Chi²
	post 2nd/3rd vaccination	34	65		7	71			
**Vaccination after hepatitis**	no	37	71		7	100		.100	Chi²
	yes	15	29		0	0			
**Vaccine post hepatitis**	mRNA	8	53		0	0			
	vector	6	40		0	0			
	protein-based	1	78		0	0			
**Medications for vaccine side effects**	no	26	50		6	86		.094	Chi²
	yes	4	8		1	14			
	unknown	22	42		0	0			
**Autoimmune comorbidities**	no	30	58		6	86		.341	Chi²
	yes	17	33		1	14			
	unknown	5	10		0	0			
**AIH simplified diagnostic score**				6 (3; 8)			7 (3;9)	.497	Mann-Whitney U
**Biopsy scan**									
**Interface**				1 (0; 4)			3 (1; 4)	.017	Mann-Whitney U
**Confluent**				2 (0; 6)			1 (0; 5)	.441	Mann-Whitney U

**Lobular**				3 (0; 4)			2 (2; 3)	.483	Mann-Whitney U
**Portal**				2 (0; 3)			2 (2; 3)	.049	Mann-Whitney U
**Ishak**				9 (0; 14)			10 (6; 11)	.757	Mann-Whitney U
**Simplified AIH**	typical	9	17		5	71		.006	Chi²
	compatible	32	62		2	29			
	atypical	11	21		0	0			
**New histological criteria**	likely	34	65		7	100		.175	Chi²
	possible	13	25		0	0			
	unlikely	5	10		0	0			
**Predominant histology**	lobular	42	81		3	43		.010	Chi²
	portal	6	12		4	57			
	others	4	8		0	0			
**Predominant histology - subtype**	confluent necrosis	30	58		3	43		.180	Chi²
	panlobular	7	13		0	0			
	>mild interface hepatitis	4	8		3	43			
	bland cholestatasis	2	4		0	0			
	mild interface hepatitis	2	4		1	14			
	central perivenulitis	5	10		0	0			
	minor change	1	2		0	0			
	steatohepatitis	1	2		0	0			
**Centralized autoantibody testing**									

**centralized autoantibody testing**	yes	28	54		3	43		.585	Chi²
**ANA**	present	21/28	75		2/3	67		.754	Chi²
**ANA titer**	80	4	19		0	0		.182	Chi²
	160	10	48		0	0			
	>160	7	33		2	100			
**AMA**	positivity	4/28	14		0/3	0		.483	Chi²
**AMA titer**	160	3	75		0	0			
	>160	1	25		0	0			
**SMA**	positivity	19/28	68		1/3	33		.235	Chi²
**SMA titer**	80	9	47		0	0		.009	Chi²
	160	9	47		0	0			
	>160	1	5		1	100			
**SMA pattern**	V	11	58		0	0		.000	Chi²
	VGT	7	37		0	0			
	G	1	5		0	0			
	GT	0	0		1	100			
**LKM**	positivity	4/28	14		0/3	0		.483	Chi²
**LKM titer**	80	1	25		0	0			
	160	3	75		0	0			
**Western blot**	negative for LKM1, LKM2, LKM3	4	80		0	0			
	borderline for PDH-E2 and BCKD-E2	1	20		0	0			
**Others**	positivity	9/27	33		0/0	0			
**Other type**	PCA	7	78		0	0			
	ARA	1	11		0	0			
	PCAlike	1	11		0	0			

**Other titer**	80	2	22		0	0			
	160	6	67		0	0			
	>160	1	11		0	0			
**Hep2 ANA**	positivity	25/28	89		2/3	66		.232	Chi²
**Hep2 ANA titer**	40	0	0		0	0		.023	Chi²
	80	6	24		0	0			
	160	15	60		0	0			
	>160	4	16		2	100			
**Hep2 ANA pattern***	homogen	5	20		1	33		.860	Chi²
	fine speckled	17	68		2	67			
	nucleolar	5	20		0	0			
	centromere	1	4		0	0			
**Hep2 SMA, AMA**	SMA	13	76		1	100		.839	Chi²
	AMA	2	12		0	0			
	AMA-like	2	12		0	0			
**Hep2 SMA, AMA (titer)**	80	6	43		0	0			
	160	6	43		0	0			
	>160	2	14		0	0			
**Liver LIA**	positivity	5/28	18		0/2	0		.513	Chi²
**Liver LIA**	LKM1	1	20		0	0			
	AMA M2	4	80		0	0			
**Gastro LIA**	PCA borderline	1	25		0	0			
	PCA	3	75		0	0			
**pIgG (normalized arbitrary units)**				1.2 (0.4; 2.7)			1.2 (0.4; 1.4)	.391	Mann-Whitney U
**pIgG (cut-off 1,27)**	negative	13	52		2	67		.630	Chi²
	positive	12	48		1	33			
**Treatment**									

**Treatment**	no	6	12		1	14		.833	Chi^2
	yes	46	88		6	86			
**Steroids for initial therapy**	no	0	0		0	0		1	Chi^2
	yes	46	100		6	100			
**Doses Steroids for inital therapy, prednisolone (mg/day)**				50 (10; 625)			45 (30; 50)	.277	Mann-Whitney U
**Azathioprine therapy**	no	40	87		6	100		.347	Chi^2
	yes	6	13		0	0			
**Azathiorprine dose (mg/d)**				50 (25; 150)					
**Liver values**									
**diagnosis**		**n=52**			**n=7**				
**ALT/ULN**				24.5 (3.9; 111.3)			19.7 (11.6; 52.5)	.845	Mann-Whitney U
**AST/ULN**				22.1 (3.0; 169.1)			18.6 (9.7; 42.3)	.829	Mann-Whitney U
**ALP/ULN**				1.4 (0.5; 8.2)			1.0 (0.8; 2.9)	.657	Mann-Whitney U
**GGT/ULN**				4.3 (0.4; 38.0)			3.7 (1.1; 9.4)	.683	Mann-Whitney U
**Total bilirubin/ULN**				5.7 (0.4; 34.4)			2.7 (0.8; 24.7)	.680	Mann-Whitney U

**INR**				1.2 (0.7; 3.2)			1.2 (1.0; 2.2)	.543	Mann-Whitney U
**IgG (g/l)**				16.8 (6.6; 34.7)			21.7 (8.7; 39.9)	.036	Mann-Whitney U
**IgM (g/l)**				1.2 (0.2; 5.3)			1.2 (0.9; 9.8)	.616	Mann-Whitney U
**original Hy's law**	yes	29	59		3	43		.414	Chi²
	no	20	41		4	57			
**new Hy's law**	yes	26	53		4	57		.839	Chi²
	no	23	47		3	43			
**R**				16.8 (2.3; 92.0)			16.5 (8.8; 28.2)	.981	Mann-Whitney U
**Liver injury pattern**	hepatocellular (R≥5)	48	94		7	100		.510	Chi²
	mixed (R 2-5)	3	6		0	0			
**Outcome**									
**Outcome**	spontaneous remission	4	8		1	14		.158	Chi²
	remission under immunosuppression (IS) & successful IS withdrawal	8	15		1	14			
	remission under IS & IS withdrawal is still ongoing	23	44		1	14			

	remission & IS withdrawal failure	2	4		2	29			
	improvement no remission despite treatment	11	21		1	14			
	transplantation or liver related death	1	2		0	0			
	death, non liver related	1	2		1	14			
	relapse treated with steroids after spontaneous remission	2	4		0	0			

* multiple answers possible, ** AST is missing for some patients

Abbrevations: SARS-CoV-2 Severe Acute Respiratory Coronavirus 2, COVID-19 Coronavirus disease 2019, AIH Autoimmune Hepatitis, IgG/M Immunoglobuline G/M, ULN Upper limit of normal, ALT Alanine aminotransferase, INR International Normalized Ratio, AST Aspartate aminotransferase, SMA Anti-smooth cell antibody, ALP Alkaline phosphatase, ANA Anti-nuclear antibody, LKM Liver kidney microsomal, AMA Anti-mitochondrial antibody, SLA Soluble liver antigen, PIgG Polyreactive immunoglobulin G, PCA Parietal cell antibody, IS Immunosuppression. R value: ALT/ULN divided by ALP/ULN.
